# Emerging Biosensors for Oral Cancer Detection and Diagnosis—A Review Unravelling Their Role in Past and Present Advancements in the Field of Early Diagnosis

**DOI:** 10.3390/bios12070498

**Published:** 2022-07-08

**Authors:** Vidhya Rekha Umapathy, Prabhu Manickam Natarajan, Bhuminathan Swamikannu, Johnson Moses, Sumathi Jones, Manoj Prathap Chandran, Madurai Kannan Anbumozhi

**Affiliations:** 1 Department of Public Health Dentistry, Sree Balaji Dental College and Hospital, Pallikaranai, Chennai 600 100, India; 2 Department of Periodontics, College of Dentistry, Ajman University, Ajman P.O. Box 346, United Arab Emirates; prabhuperio@gmail.com; 3 Department of Prosthodontics, Sree Balaji Dental College and Hospital, Pallikaranai, Chennai 600 100, India; registrar@bharathuniv.ac.in; 4 Department of Anatomy, Sree Balaji Medical College and Hospital, Chromepet, Chennai 600 044, India; profjohnson@bharathuniv.ac.in; 5 Department of Pharmacology, Sree Balaji Dental College and Hospital, Pallikaranai, Chennai 600 100, India; sumathijones@gmail.com; 6 Junior Research Fellow, Sree Balaji Dental College and Hospital, Pallikaranai, Chennai 600 100, India; prathapmanoj12@gmail.com; 7 Department of Pathology, Sree Balaji Medical College and Hospital, Chromepet, Chennai 600 044, India; anbumozhi.m@bharathuniv.ac.in

**Keywords:** biosensors, oral cancer, biomarkers, saliva, electrochemical biosensor, optical biosensor, nano biosensor

## Abstract

Oral cancer is a serious concern to people all over the world because of its high mortality rate and metastatic spread to other areas of the body. Despite recent advancements in biomedical research, OC detection at an early stage remains a challenge and is complex and inaccurate with conventional diagnostics procedures. It is critical to study innovative approaches that can enable a faster, easier, non-invasive, and more precise diagnosis of OC in order to increase the survival rate of patients. In this paper, we conducted a review on how biosensors might be an excellent tool for detecting OC. This review covers the strategies that use different biosensors to target various types of biomarkers and focuses on biosensors that function at the molecular level viz. DNA biosensors, RNA biosensors, and protein biosensors. In addition, we reviewed non-invasive electrochemical methods, optical methods, and nano biosensors to analyze the OC biomarkers present in body fluids such as saliva and serum. As a result, this review sheds light on the development of ground-breaking biosensors for the early detection and diagnosis of OC.

## 1. Introduction

The growth of malignant tissue in the oral cavity, which usually affects the tongue, floor of the mouth, cheek, gingiva, lips, or palate, is recognised as oral cancer (OC). OC accounts for one-third of all cancer cases worldwide and India accounts for roughly 30% of all cases [1]. The most frequent indicators of OC are a non-healing sore in the mouth and discomfort that is difficult to ease. Other symptoms include a lump or thickening in the cheek; white or red spots on the gums, tongue, and other regions of the oral cavity; as well as a persistent sore throat and difficulty eating or swallowing. OC can be avoided by reducing the risk factors such as consumption of tobacco (both smokeless and chewable) and alcohol, as well as raising awareness about these issues. All of these variables contribute to the commencement of oral squamous cell carcinoma (OSCC) by producing genetic and epigenetic alterations. The oral aetiology begins with a burning feeling in the mouth, some restriction while opening the mouth, and any red or white lesions in the oral cavity; however, a lesion’s histology may show dysplastic characteristics. Such conditions are categorised under oral potentially malignant disorders (OPMDs). Early identification is critical for the disease’s prognosis and the sufferer’s survival if not detected in the very early stage of OPMD, which can be life-threatening and associated with increased morbidity.

In addition to the high mortality rate, OC is a major contributor to lower productivity in developing countries due to early deaths [2,3]. Visual inspection and biopsy are the most common procedures for diagnosing OC and premalignant lesions (e.g., leukoplakia and erythroplakia). Currently, OC is diagnosed using invasive methods such as tissue biopsy of the infected area, followed by non-invasive medical imaging techniques that are both costly and time-consuming [4,5]. Traditional methods of distinguishing OSCC from normal oral mucosa have been exploited individually or in combination with an adjuvant investigation strategy. These approaches are intrusive, time-consuming, costly, labour-intensive, and reliant on the investigator’s competence. The condition may go unnoticed until a particular diagnostic test is performed because of low amounts of biomarkers in exfoliated cells, tissue samples, or biological fluids (saliva, human blood, semen, and urine). The quantity of these markers is minimal in a healthy person but it increases as the disease advances.

Biosensors are receptor–transducer devices that use biological material to interact with an analyte [6] and deliver quantitative or semi-quantitative information utilising a biological recognition element. Biosensors are popping up all over the place in the medical profession. They are employed as diagnostic instruments for identifying infections, monitoring and detecting hazardous metabolites, glucose monitoring, cholesterol testing, and vitamin and other nutrient measurements [7,8,9] Oral fluid-based biosensors, for example, are used in dentistry to diagnose caries, periodontitis, and oral cancer by detecting saliva and GCF samples [10]. This article reviews the developments and advancements in different biosensors used in the early detection of OC and OSCC. We also explain the methods used by biosensors to target different types of biomarkers and focused on biosensors that function at the molecular level.

## 2. Requirement of Biosensors in Diagnostics

Biosensor development is critical for investigating the biomarkers responsible for these malignancies and diagnosing OC in its early stages [11]. When cancer is identified early, the patient’s prognosis and chances of survival will improve. This necessitates immediate consideration of the accessibility of non-invasive, patient-friendly diagnostic technologies that are minimally or entirely non-invasive. Biosensors can precisely and accurately assess the number of biomarkers, assisting in the right diagnosis of OSCC development. As a result, early diagnosis of OSCC is essential for disease treatment and improved well-being. Progressions in emerging biosensor-based diagnostics and screening devices have opened the path for OC or OSCC detection assays that are rapid, simple, accurate, and robust.

Emerging research on biosensors has resulted in the establishment of diagnostic instruments with increased sensitivity and turnaround time. These technologies surely assist physicians in the early detection and treatment of a variety of diseases. Biosensors have several applications in the medical field, such as monitoring glucose levels in diabetic patients, detecting pathogens and toxic metabolites, as well as measuring folic acid, biotin, vitamin B12, and pantothenic acid [7,8]. However, this technology is still emerging in dentistry. With the availability of these devices, diagnostic tests can be rapidly performed within clinics.

A biosensor, by definition, is a self-contained analytical instrument that includes a biologically active substance in close contact with an appropriate transduction element for the goal of detecting (reversibly and selectively) the concentration or activity of chemical species in any type of sample [12]. Clark and Lyons created the first biosensor, an enzyme-based glucose sensor [9]. These instruments are more precise, have a faster reaction time, and can detect nonpolar compounds.

**Figure 1:**
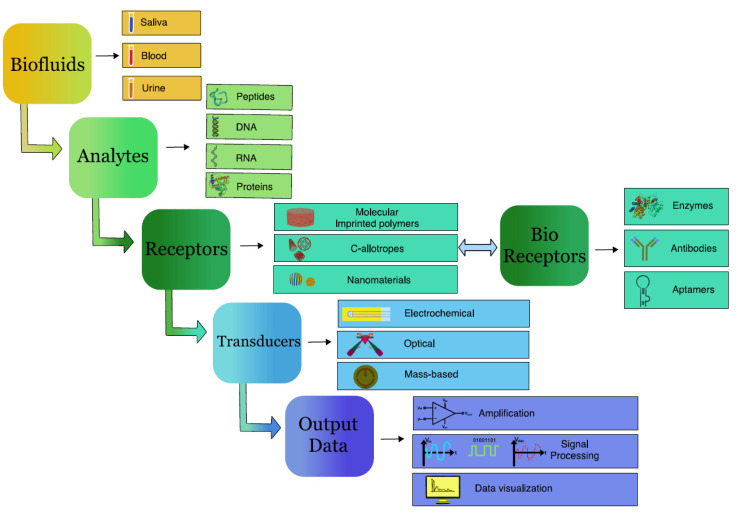
Basic components of biosensors.

Because of the non-invasive early diagnosis of cancer, researchers all over the world have begun to design and develop biosensors that can effectively detect cancer. Biosensors are devices that transform a biological entity (protein, DNA, or RNA) into an electrical signal that can be detected and processed in order to detect a specific biological analyte [13]. The term “bio” is used since the sensor detects biological elements such as DNA, RNA, protein, antibodies, microorganisms, etc. A biological identification element, a transducer, and a signal processing system are the three most significant components of a biosensor (Figure 1) [14]. The biological material must have a high level of specificity, stability, and immobilisation. Biosensors are divided and categorised based on the biorecognition element, detection technique, and kind of interaction used for bio-analyte detection. Based on the biological recognition element, they are categorised into enzymatic, protein, receptor-based, DNA, and whole-cell biosensors [15]. When the target bio-analytes are in close proximity to the biorecognition element, the functioning concept is to recognise them in the bio-fluid. The biosensor’s sensitivity, precision, reproducibility, stability, and robustness are all controlled by biomolecules or biomimetic materials immobilised on the substrate’s surface. When a target bio-analyte interacts with an immobilised probe, electrical/fluorometric/luminometric/colourimetric signals are generated depending on the presence or absence of the target bio-analyte in bio-fluid. The recognition signal events are converted into electrical impulses by the transducer, which converts them into a measurable form. Biosensors are further categorised into the following groups based on the signal transduction mechanism: electrochemical, thermal, optical, or mass-sensitive [15]. Although electrochemical transducers are the most common in sensor technology, optical transducers are gaining popularity in the present day due to their numerous benefits. The signals from the transducer are amplified and displayed by an electrical system.

## 3. Various Types of Biosensors Used in Detection of Cancer

Biosensors used for identifying cancer indicators are being designed and developed by researchers and scientists in order to detect early cancer. OC may be detected effectively and early using biosensors. DNA, RNA, and protein biosensors have all been proven in studies for their efficiency in detecting OC and providing useful information to allow for non-invasive OC detection [16]. The use of biosensors for protein biomarker analysis has emerged as a promising and cost-effective method for developing point-of-care devices [17]. Electrochemical biosensors have been used in detecting cancer markers [18]. Surface plasmon resonance sensors (SPR), which are based on spectroscopy of surface plasmons, are being employed for the label-free detection of cancer markers [19]. Because of their light weight, great sensitivity, and low power requirements, piezoelectric biosensors have also been used to detect cancer markers [20]. Tan et al. developed a surface-immobilised optical protein sensor to detect an IL-8 marker for the diagnosis of OC [21]. Yuan et al. created an SPR-based biosensor for detecting cancer markers in ovarian cancer patients [22]. Kumeria et al. created a microchip biosensor for detecting circulating tumour cells based on nanoporous aluminia [23]. Malima et al. have developed a very sensitive microscale in vivo sensor for various biomarker detections that is enabled by the electrophoretic assembly of nanoparticles [24]. Because of their excellent sensitivity, specificity, compact size, rapidity, and cost-efficiency, optical biosensors are currently gaining popularity for biomarker detection [25]. Nanotechnology, MEMS (micro electromechanical systems), NEMS, biotechnology, and other multidisciplinary techniques have been applied in the development of novel optical biosensors.

### 3.1. DNA Biosensor

DNA is the genetic information carrier and the building block of biological heredity. Following the identification of DNA, DNA-based diagnostics such as the RAPD, RFLP, and PCR methods emerged. About 5–10% of malignancies are inherited and caused by single-gene mutations. Several hereditary cancer syndromes and their causative genes have been identified. In order to improve patient care, molecular-based laboratories have been involved in the development of innovative tests that are reliable, affordable, and low-cost. They are also utilised to improve current procedures by making them faster and more cost-effective. Molecular diagnostics has proposed a very sensitive and quantitative technique for detecting disease-causing pathogens and genetic variations based on genomic sequence analysis (Table 1). Many DNA testing methods have been developed as a result of the efforts of researchers.

The identification of DNA sequences and the discrimination of sequences is difficult and time-consuming and has low hybridisation efficiency. To address these issues, DNA sensors were included in high-throughput analysis, implying a significant reduction in effort, time, and cost. Biosensors are biorecognition elements that, when combined with various transduction mechanisms, have aided the rapid expansion in the domain of bioanalysis and its associated technologies [26,27,28]. These characteristics, as well as additional benefits such as the ease of manufacturing and operation and the cheap costs, make it an attractive option for the non-invasive early detection of OC in saliva.

### 3.2. RNA Biosensor

Multiple cancer-causing defects, such as the inactivation of the anti-tumour gene, chromosomal degradation, and gene hypermethylation, change the signature of normal cells. These cancer-causing aberrations, such as microRNAs (miR), are considered RNA-based cancer biomarkers. RNA biomarkers allow cancer to be diagnosed even when no physical signs are present. Wang et al. produced a POC adaptable magnetic-controllable electrochemical-based biosensor with great sensitivity [18]. It shows an early-stage OC biomarker (miR) diagnosis with a limit of detection (LOD) of 0.22 aM (2.2–19 M). The various RNA biosensors used for the detection of cancer are summarised in Table 1.

Luo et al. discovered a ratiometric electrochemical biosensor based on a locked nucleic acid (LNA)-aided strand displacement process with greater repeatability for detecting exosomal miR-21 originating from cancer with a LOD of 2.3 fM [29]. They created a Y-like structure helped by LNA that activates in the presence of miR-21 as a target biomarker, with detection validated by electrochemical impedance spectroscopy (EIS) and DPV. To detect miR-21, Sabahi et al. created a biosensor based on a dendritic Au nanostructure grafted with single-wall carbon nanotubes (SWCNTs) and a modified fluorine-doped tin oxide (FTO) electrode [30]. MiR-12 was used as a specific biomarker for different cancers in the range of 0.01 fmol L^−1^ to 1 mol L^−1^, with a detection limit of 0.01 fmol L^−1^.

### 3.3. Protein Biosensor

For POC and clinical analysis, electrochemical biosensors provide a sensitive, fast, and low-cost diagnostic framework for detecting protein cancer biomarkers [31]. The surface of the electrodes in these biosensors is often modified with receptors such as antibodies or aptamers. Because the interactions between an antibody and an antigen operate similar to a lock-and-key binding mechanism, immunosensors are very selective [32]. Protein biomarkers for cancer detection can be used to measure components that are thought to be indications of aberrant biological processes, disease processes, or treatment intermediation responses [28,33]. These biomarkers are frequently collected from biofluids and their expression level usually indicates disease status. Since many tumour markers are identified in saliva, the salivary samples are of great interest for the non-invasive screening of OC [34,35]. Research from Markopoulos and co-workers has revealed that both cell-free mRNAs and proteins in saliva have diagnostic relevance for OC [36]. Wei et al. were pioneers in the development of multiplexed electrochemical sensors for the measurement of salivary biomarkers for the diagnosis of OC [32]. Their findings revealed that the multiplex detection of IL-8 (both mRNA and protein) allowed for a precise OC diagnosis. Human saliva samples were utilised in another investigation to create electrochemical magnetobiosensors for the detection of both mRNA and the protein IL-8 [25]. Highly sensitive and selective techniques were revealed in this study. The enzyme-linked immunosorbent test (ELISA) is the gold standard approach for clinical biomarker detection [37]. For many protein analytes, the limits of detection (LOD) of ELISA are 1–3 pg mL^−1^ [28,38]. ELISA, on the other hand, is restricted by the expense of the test kits and equipment, the length of time it takes to measure, and the difficulty of multiplexing. As a result, when it comes to POC diagnostics, ELISA is not the first option.

Aptamer-based biosensors have label-free and high sensitivity for electrochemical detection when compared to standard biosensors [39,40]. A capacitive aptasensor was created to track the overexpression of the human epidermal growth factor receptor 2 (HER2) protein, which has been linked to ovarian, lung, stomach, and oral malignancies [41]. On a gold microelectrode surface, anti-HER2 aptamers (ssDNA) are immobilised. Then, various doses of HER2 were spiked in diluted human serum. The link between capacitance and the HER2 concentration was finally determined [39]. Aptamers were also used to identify the IL-6 protein in which EIS was used to develop a nano-aptamer sensor to detect IL-6 in biofluids [42] and 0.02 pg mL^−1^ was considered to be the detection limit. Furthermore, after two weeks, the aptasensors had 90% of their original impedance response signal, indicating the sensor’s great stability.

**Figure 2:**
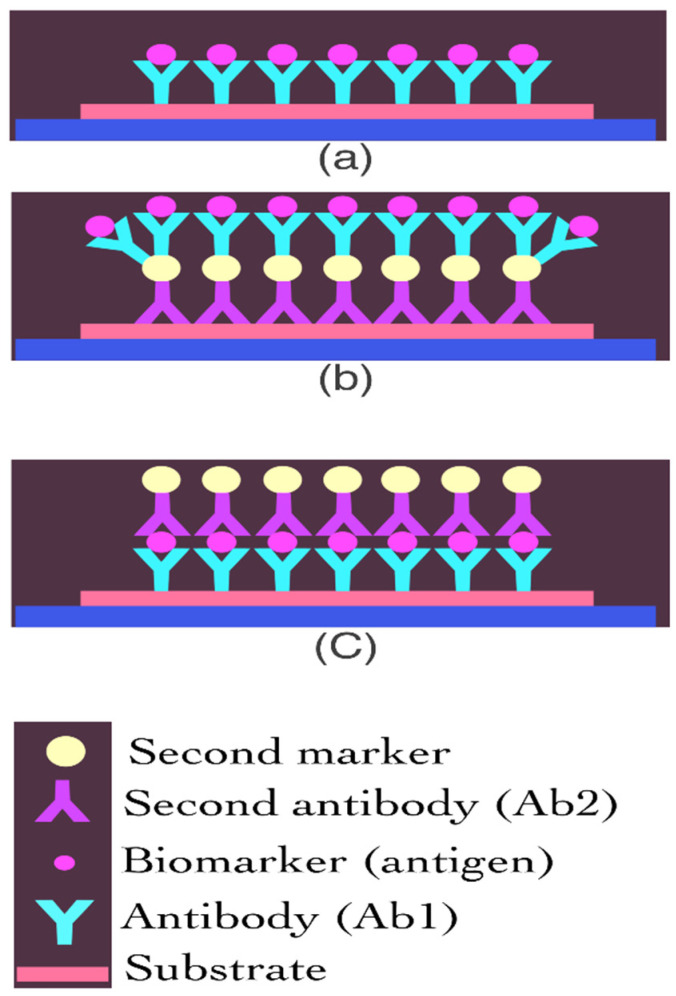
Schematic representation of an immunoassay-based protein biosensor. (**a**) Label-free biosensor; (**b**) and (**c**) Labelled biosensor.

Immunoassays are another type of protein sensor. A recognised element, such as a primary antibody, is immobilised on an electrode surface in a sandwich immunoassay to capture the specific analyte. Then, to bind the antigen, a secondary antibody coupled with an enzyme, such as horseradish peroxidase (HRP), is added to the solution (the analyte). HRP can convert its substrate to electrochemically active species, allowing it to translate chemical signals into electrochemical signals (Figure 2) [25,43,44]. Heineman’s group, for example, was a pioneer in enzyme-linked electrochemical analyte detection utilising sandwich immunoassays [45]. For multiplex electrochemical protein identification, two primary techniques are used in this study. The analytes were first bound to the main antibody and then secondary antibodies were added to the nanoparticles. Second, electrodes were used to immobilise and measure the electrochemical signals after being immobilised with different antibodies. Bioreceptors, redox mediators, and transducer networks are all used in the development of sensitive and accurate electrochemical biosensing frameworks. Nanomaterials have been increasingly popular in recent years, and their high surface-to-volume ratio has led to their widespread use in the biosensor field. New carbon or metal nanomaterials, such as graphene and its derivatives, have been developed with unique qualities, such as high electron transfer rate and biocompatibility, and as a result, numerous studies have combined nanomaterials with immunosensors [25,46,47,48,49]. When compared to flat surfaces, nanostructure-based immunosensors with an increased electrode surface area can provide 10-fold or greater antibody coverage, resulting in up to 1000-fold increased sensitivity and a lower limit of detection [50,51,52].

## 4. Oral Fluids as Biomedia for Diagnostics

Blood and urine are the most frequent media utilised in regular laboratory procedures. They include a variety of biological compounds that aid in disease diagnosis. The disadvantages of utilising blood as a biomedia include intrusive sample collection, patient fear and anxiety, and the danger of disease transmission. The other medium is urine, which is difficult to collect, especially in mobile people. As a result of these constraints, there was a higher need for alternate biomedia. Oral fluids (gingival crevicular fluid [GCF] and saliva) are easily accessible biofluids that can provide a rapid and non-invasive sample without requiring any medical expertise. Because the procedure is non-invasive, patients who require regular monitoring can have samples taken an unlimited number of times. When compared to urine drug testing, most of its contents are similar to serum and it can identify drugs promptly. Oral fluids have all these characteristics making them a promising diagnostic medium [53].

Saliva contains a variety of electrolytes, including K, Mg, Ca, Na, and P as well as biological elements such as proteins, enzymes, immunoglobulins, and nitrogenous products. Lubrification, antimicrobial activity, buffering action, digestion, and tooth protection are the key activities of this biofluid [54,55]. Saliva has piqued the interest of the scientific community, which has resulted in a flurry of new research. Saliva has been employed as a diagnostic tool in a variety of sectors, including pharmacology, medicine, and dentistry [56]. The term ‘salivaomics’ refers to research on the saliva’s genome (genomics), RNA (transcriptomics), metabolite profiles (metabolomics), proteins (proteomics), and microbial community (microbiomics). Among them, the investigation of DNA methylation, a stable epigenetic change that has been linked to systemic diseases including chronic renal disease progression and respiratory allergies [57,58,59], is included in research on the salivary genome and epigenome. Another useful approach for diagnosing some systemic disorders is the identification of particular microRNA segments. In fact, a subset of miRNA sequences has been frequently identified in schizophrenia patients [60,61].

**Table 1:** Types of biosensors categorised based on the biological recognition elements.

Sl. No	Biosensor	Method	Source	Advantage	Reference
DNA biosensor					
1	Immobilisation-free, ultra-high selective electrochemical biosensor	Nicking endonuclease-aided target recycling	Saliva	High specificity and good discrimination at single-base mismatch.	[28]
2	Robust ratiometric electrochemical DNA biosensor	Exo III-assisted target recycling	Saliva	Detect low concentration of biomarkers	[62]
3	Detection of oral cancer overexpressed 1	Nuclease-assisted target recycling and DNAzyme	Saliva	Ultra-high discrimination capability with single-base mismatch detection	[63]
4	Biocompatible DNA dendrimer system	Modified short nanometer of DNA working on the electrode surface	Saliva	Can detect multiple biomarkers at same time	[64]
RNA Biosensor					
5	Magnetic controllable electrochemical biosensor	miRNA	Artificial saliva	High sensitivity, detect cancer at early stage	[38]
6	Ratiometric electrochemical biosensor	Locked nucleic acid	Exosomes	Detect exosomal miR-21 with LOD 2.3 fM	[29]
7	Single-wall carbon nanotubes	Dendritic Au nanostructure modified fluorine-doped tin oxide	Serum	High sensitivity with LOD 0.01 fmol/mL	[30]
Protein Biosensors					
8	Multiplexed electrochemical sensors	Detection of salivary biomarkers	Saliva	Multiplex detection of protein and mRNA IL-8	[32]
9	Capacitive aptasensor	Detection of HER2 protein	Serum	Determine the link between capacitance and HER2 concentration	[39,41]
10	Nano-aptamer sensor	Detect IL-6	Sweat	Detect low concentration at 0.02 pg/mL	[42]

## 5. Saliva-Based Biosensors

Saliva is a thin, watery liquid secreted by the salivary glands into the mouth. Active transport or passive diffusion can bring salivary components from salivary glands or the associated vasculature. Proteomic, microbiome, immunologic, genomic (transcriptomic and epigenome), and metabolomics biomarkers have been identified as components that correlate with specific disorders. Three groups of researchers in the United States have successfully discovered 1166 proteins in human saliva. Matrix metalloproteinases (MMP1, MMP3, and MMP9), cytokines (IL-6, IL-8), and vascular endothelial growth factor were among the biomarkers they discovered. Tumour necrosis factor-alpha and salivary transferrin have also been identified as possible biomarkers for OC diagnosis because of the direct interaction between saliva and OC lesions. On the other hand, salivary soluble CD44 Ag can be used as a biomarker for head and neck squamous cell carcinoma [65], whereas OSCC-related salivary biomarkers include Cyfra 21-1 [66,67], tissue polypeptide Ag, cancer Ag 125 [66], and salivary zinc finger protein 510 peptide [68]. Saliva has recently been proved to be a useful diagnostic tool for diseases including human immunodeficiency virus (HIV) and hepatitis A, B, and C, in which immunologic markers such as IgG and microbics play a vital role. Periodontitis and Sjögren syndrome have been linked to a number of salivary metabolics. Genetic variations in patients can be found by examining micro-RNA (mi-RNA) markers. As a result of these indicators, saliva appears to be a viable diagnostic fluid [69,70]. However, there are a number of downsides to using saliva as a diagnostic fluid, the most significant of which is its low specificity and sensitivity. Due to the lack of a consistent quantity of saliva in people, the concentration of analytes might vary substantially depending on when the sampling/collection method is performed [55]. However, the lower level of analytes in saliva is no longer a restriction because many new and sensitive methods, such as microfluidics and nanotechnologies, are introduced, which have enhanced sensitivity and assay speed. Microfluidics and microelectromechanical devices for DNA, gene transcripts (mRNA), proteins, electrolytes, and tiny chemicals in saliva, as well as the overall profile, correlate to a disease state. These technologies identify diseases using a mix of many biomarkers rather than a single biomarker, addressing the limitations of sensitivity and specificity in single-marker locations [71].

### 5.1. Salivary Metabolomics

Saliva has advantages over other biofluids, such as blood and urine, since its collection is non-invasive and relatively fast. Some salivary metabolites have been successfully identified using 1 H NMR [72,73,74,75,76,77], and inter-subject variability has been studied [75,76] but to date, chemometrics has not been applied to saliva metabolite datasets to find the potential markers for disease. As a first step in understanding the human saliva metabolome, we employed high-resolution 1 H NMR spectroscopy to determine if the salivary metabolite composition differs due to gender, stimulation, or smoking status. Takeda and co-workers established the composition and concentration of salivary metabolites in a normal human population and how health choices, such as smoking, may affect the metabolic profile [78].

### 5.2. Salivary Proteomics

A global quantitative analysis of human salivary proteins without resources using a mass spectrometer can be easily obtained using a 2-DE strategy, which is widely used for biomarker discovery, namely for oral diseases, dental caries [79] and periodontitis [80], and also for other pathophysiological conditions such as Sjögren syndrome (SS) and non-Hodgkin’s lymphoma [81]. In addition to biological variability, technical bias induced by protein migration during the focusing step or by gel staining could make it difficult to spot detection and their boundaries, making 2-DE gel analysis a hard task. Regarding salivary peptidome, efforts have been made using label-free quantitation to evaluate the expression of the major salivary peptides (statherin, cystatins, PRPs, SMR3B (P-B peptide), and histatins) under different conditions.

## 6. Electrochemical Biosensors

**Figure 3:**
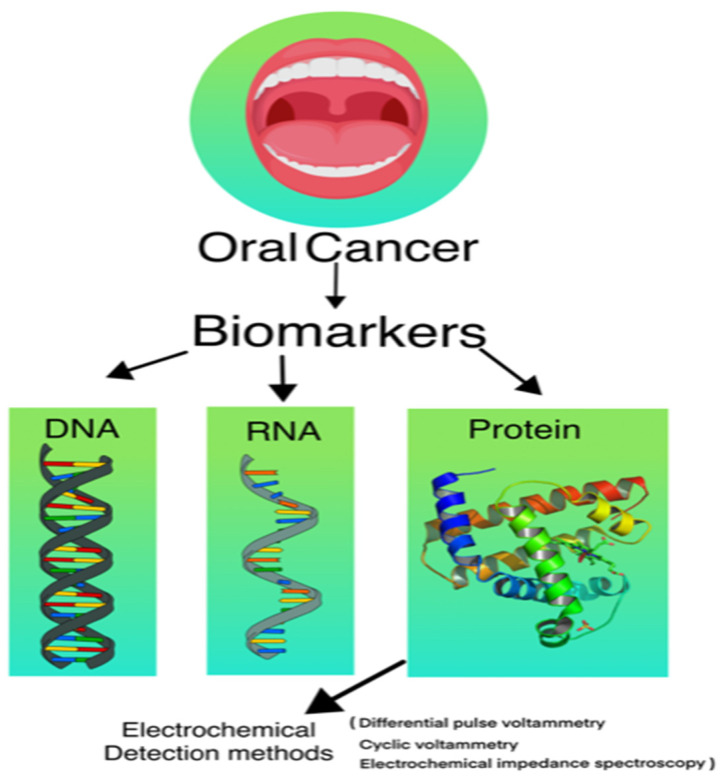
Schematic representation of electrochemical-based detection of biological biomarkers.

Electrochemical biosensors have a higher rate of implementation than other biosensor technologies because they can detect practically any type of biomarker and are simple to combine with typical laboratory benchtop equipment (Figure 3). Furthermore, because they are easily downsized, they are likely to be integrated into wearable and portable devices [82,83,84,85]. The integration of electrochemical sensors into compact devices must meet strict requirements for convenience, comfort, ease of operation, and flexibility, making the creation of dependable, wearable, and portable POC ultrasensitive devices difficult. To functionalise the surface of the sensing electrodes, a variety of methods have been explored including antibodies, magnetic beads, and aptamers (Table 2). Researchers have identified many possible biomarkers for OC [86]. One of them is the cytokeratin family member, Cyfra21.1, a fragment of cytokeratin-19 that has been widely studied in saliva using biosensors.

## 7. Optical Biosensors

Optical devices offer a new way to conduct salivary analysis with great sensitivity and selectivity, and without the need for labels. Fluorescence-based biosensors, surface-enhanced Raman spectroscopy biosensors, photonic crystal biosensors, and surface plasmon resonance biosensors are among the non-invasive optical technologies that have been developed [87]. Colourimetry-based biosensors are also known as optical sensors and they provide a high-quality and quick technique to perform non-invasive diagnostics of biofluids. Microfluidic channels are commonly found in optical systems, ensuring that biofluid is transported to the region where the detector/reader is positioned for a precise and reproducible examination [88]. Due to its high sensitivity, adaptability, simplicity, and multiplexing capabilities, SERS is the most often-used analytical technique among the optical methods [89]. In this case, nanostructured materials are frequently used to improve the device’s sensitivity.

## 8. Nano Biosensors

Biosensor-based detection has several advantages over traditional approaches, including affordability, simplicity of handling, miniaturisation, and the lack of the need for an expert to analyse the data. Fabricated biosensors are found to have some flaws such as low sensitivity, instability, and other issues [90]. As a result, integrating developments in biosensing with nanotechnology aids in overcoming the aforementioned constraints and provides a considerable improvement in strategies for the detection and diagnosis of OSCC. In this context, gold nanoparticles (AuNPs), quantum dots (QDs), dendrimers, metal oxides, carbon-based nanocomposites, and other nanomaterials (NMs) have been employed to fabricate nanobiosensors. Biosensor functionality has increased as a result of the unique features of the aforesaid NMs, with quicker detection, improved detection limits, and enhanced repeatability [91,92]. Because of these improvements, these nanobiosensors require a smaller sample volume and offer high precision (less than 1% error rate), specificity, cost-effectiveness, and reduced time and ease of implementation for automatic analysis. As a result, it is suggested that using nano-based biosensing for the early identification of OC could lead to enhanced patient treatment outcomes and care. Nanotechnology-based miniaturised devices are a breakthrough in pre-clinical and clinical research fields including medication delivery, customised medicine, and diagnostic potential, a field now known as “nanodiagnostics”.

Advancements in nanotechnology offer the necessary impetus for the development of nanobiosensor technologies [93,94]. Individual/singular optimisation is required for characteristics such as specificity, sensitivity, shelf life, linearity, detection limit, reaction time, and repeatability, which influence the performance of a nanobiosensor [95]. Because of the higher relative surface area and quantum confinement effect, NM-based scaffolds greatly improved the aforementioned metrics when compared to bulk equivalents. NMs also have a high surface-area-to-volume ratio, which increases chemical reactivity and stability. These scaffolds can accommodate many ligands due to their high surface-to-volume ratio, thereby boosting binding affinity and increasing selectivity. The quantum confinement effect, on the other hand, confines the mobility of randomly moving electrons at a certain energy level as the particle size decreases until it reaches the nano range. This confinement causes a rise in the band gap and a decrease in wavelength, which are critical for influencing the characteristics of the materials. Along with the basic NM features, shape (nanospheres, nanotubes, nanowires, and so on) and size (nanospheres, nanotubes, nanowires, and so on) play a key role in influencing their behaviour [96,97,98,99,100]. The biosensing behaviour of NMs has been shown to improve when their particle size is reduced [101]. Clinically, determining the initial stage of OPMDs or OSCC solely from a single biomarker is difficult. As a result, multiplexing methods incorporating the detection of numerous biomarkers are necessary for better prediction. Immobilising different biorecognition probes specific to the target onto the surface of NMs could enable this multiplexed detection. This method has been successfully tested on a clinical platform for minimum or entirely non-invasive OSCC detection [45,102].

When compared to a standard sandwich ELISA system, the addition of 3DN-CNTs increased the sensitivity of a developed biosensor by almost twenty times [103]. Upconversion nanoparticles (UCNPs) [104,105], which display photon upconversion phenomena when activated by incident near-infrared (NIR) light with emission in the visible region of the electromagnetic spectrum, are other prominent types of NMs in biosensing. It has been reported that biocompatible UCNP composites based on fluorescence resonance energy transfer (FRET) may detect OSCC biomarkers using energy changes in the red and blue wavelength ranges. The expression of matrix metalloproteinase 2 (MMP2) in tumour models and OSCC tissues could be detected using these UCNP composites. The developed nanocomposite generates FRET-induced red fluorescence when irradiated but emits blue fluorescence when MMP2 is present [105], indicating their potential for clinical diagnosis in OSCC.

**Table 2:** Various biosensors used in the detection of OC.

Type	Biomarker	Detection Limit	Source	Advantage	Reference
	Electrochemical biosensors				
Electrochemical sandwich-type immunosensor	Interleukin 1 (IL-1)	5.2 pg/mL	Saliva	Time to obtain results is faster compared to ELISA	[106]
Immunosensor by immobilising anti-Cyfra21.1 on a gold electrode modified with cysteamine and glutaraldehyde	Cytokeratin Cyfra21.1	2.5 ng/mL	Saliva	Low-cost, dependable, and robust approach for detection of non-invasive salivary Cyfra21.1	[107]
Label-free immunosensor	Interleukin 1 (IL-1)	7.5 fg/mL	Serum and saliva	6-phosphonohexanoic acid (PHA) is used as a biomolecule immobilisation matrix.	[108]
Magnetic beads-based electrochemical biosensor	Hypoxiainducible factor-1 alpha (HIF-1)	76 pg/mL	Saliva	The biosensor was built in a sandwich shape to require less incubation stages, resulting in a shorter total test time compared to traditional laboratory methods.	[109]
A ratio-metric electrochemical sensor	Oral Cancer Overexpressed 1 (ORAOV1)	12.8 fM	Artificial saliva	This method is to overcome the limitations of traditional electrochemical biosensors with signal-on/signal-off outputs.	[62]
Dual SPCE-based immunosensor	IL-1 and TNF	0.38 for IL-1 and 0.85 for TNF	Serum and saliva	Multiplex and sensitive amperometric biosensor with very low costs.	[110]
SiNW sensor array (Silicon nanowire)	TNF- and IL-8	100 fg/mL	Saliva	Uses intrinsic opposing charge to enable straightforward differentiation	[45]
	Optical Biosensors				
Fluorescent immunosensor	Cyfra21.1	0.5 ng/mL	Clinical saliva	The 3DN-CNT sensor enhances the sensitivity of Cyfra 21-1 detection by increasing the density of immobilised antibodies through its high surface area.	[111]
Microfluidic biosensor	IL-8, IL-1, and MMP-8	80 pg/mL	Saliva	Multiplexed detection of salivary biomarkers	[112]
Fluorescent biosensor with magnetic and fluorescence bioprobes (MFBPs)	CD63 proteins	Lower than 500 particles/mL	Saliva	One-step quantification with less assay time; achieved high sensitivity with low limit of detection	[113]

## 9. Conclusions

Because of the limitations of existing cancer detection techniques, cancer researchers and scientists are focusing on the development of biosensors for the efficient and quick non-invasive detection of cancer indicators. Cancer markers are substances that demonstrate the presence of cancer cells in the body. These indicators can be found in blood, saliva, or other bodily fluids. Most previous studies are based on a technological developmental approach involving few subjects and thousands of different variables. In order to translate this basic information into clinical practice for the diagnosis and prevention of oral cancer, including various clinical applications, new transitional and translational studies are needed. Properly planned and designed clinical trials on the diagnostic, predictive and prognostic performance of biosensors will pave the path for their inclusion in regular clinical practice. The biosensors that have been reported in the review should be analysed in future studies for their applicability in routine clinical practice. Novel biomarkers for various cancers are being continuously researched and a few have been clinically employed for cancer screening and monitoring. The development of biosensors paves the way for a new and innovative approach to the speedy early identification of cancer. It will be difficult to convince clinicians without substantial validation due to limited studies supporting the sensitivity and specificity of biosensors, and it is highly unlikely that clinicians would be willing to include biosensing tools in clinical practice, given the possibility of numerous false-positive and false-negative results. As we all know, the gold standard laboratory techniques require specialized equipment and trained personnel in order to operate the systems and equipment. This reflects the higher costs associated with the analysis the increased length of time to realize the outcomes. Advanced biosensing systems can be manufactured at relatively cheaper costs compared to laboratory equipment. Due to the direct interaction of saliva with premalignant or malignant lesions, OC is mostly treated using wearable sensors inserted into the mouth cavity. In the future, wearable intraoral bioelectronic platforms and portable POC devices are expected to have increased application in clinical investigations and diagnoses as they offer substantial advantages compared to traditional laboratory equipment and procedures.

## Data Availability

Not applicable.
